# Loneliness and Depression among Women in Poland during the COVID-19 Pandemic

**DOI:** 10.3390/ijerph182010698

**Published:** 2021-10-12

**Authors:** Anna Idzik(199), Anna Leńczuk-Gruba, Ewa Kobos, Mariola Pietrzak, Beata Dziedzic

**Affiliations:** Department of Development of Nursing, Social and Medical Sciences, Medical University of Warsaw ul., Żwirki i Wigury 61, 02-091 Warsaw, Poland; anna.lenczuk-gruba@wum.edu.pl (A.L.-G.); ewa.kobos@wum.edu.pl (E.K.); mariola.pietrzak@wum.edu.pl (M.P.); beata.dziedzic@wum.edu.pl (B.D.)

**Keywords:** loneliness, depression, anxiety, COVID-19, women

## Abstract

Background: The COVID-19 pandemic has forced many changes in the functioning of people all over the world in a short period of time. According to a WHO report (2020), it is women who are at a particular risk of the negative effects of the pandemic, especially in terms of mental health. Aim of study: The aim of the study was to assess the prevalence of anxiety, depression, irritability, and loneliness among adult women during the COVID-19 pandemic. Materials and methods: The study was conducted on a representative sample of women in Poland (n = 452). The data were collected using the HADS-M scale and the R-UCLA scale. Results: A low level of loneliness was found in 37.3% of the women, moderate in 38.9%, moderately high in 22.3% and very high in 1.3% of women. Self-rating of physical and mental health was significantly positively correlated with anxiety, depression, and irritability in HADS-M, and loneliness in R-UCLA. As the severity of loneliness increased, so did Hospital Anxiety and Depression Scale scores on all subscales (*p* < 0.001). Conclusions: The study group presented with mental well-being disorders in the form of anxiety and depression. Two in three women experienced loneliness.

## 1. Introduction

The outbreak of the COVID-19 pandemic caused much fear and concern regarding the course of the disease itself and its numerous potential complications [1]. The pandemic has caused many changes in the functioning of people over a short period of time. These changes are negative in the vast majority of cases. The enforcement of safety rules through legislation by the authorities of different countries to prevent the transmission of the SARS-CoV-2 virus contributed to anxiety, unpredictability, and loss of control over important life dimensions. Forced isolation and social distancing could be the cause of loneliness and exacerbated depressive symptoms among people. Despite cultural differences in the social understanding of the notion of mental health, due to the worldwide reach of the pandemic, one may assume that it is a collective experience affecting mental health regardless of the geographical region. The pandemic causes justified anxiety in the majority of people [2]. Social isolation can involve a lack of contact or physical separation from one’s family, friends, or wider social networks [3,4].

As research conducted in Poland [5] and other countries [6,7] has shown, the sense of loneliness experienced during the current pandemic is usually associated with limited social contact. Thus, the possibility of entering social roles in relation to one’s needs has become limited, contributing to anxiety and depression. It seems that in the face of the threat posed by the COVID-19 pandemic, exacerbation of depressive symptoms or increases in emotional tension are a natural response of the body to major changes, uncertainty and threats faced by society [8].

Jia et al. emphasized the urgent need for evidence of mental health problems during the COVID-19 pandemic to identify those at highest risk and to investigate psychological and social resources that can mitigate this risk [9].

On the other hand, the COVID-19 epidemic and its economic and social consequences can exacerbate depressive symptoms to a clinically significant extent in some individuals. Loneliness is one of concomitant symptoms in patients with depressive disorders. For this reason, depressive disorders are associated with loneliness [10]. Neurobiological research on the impact of loneliness on health shows that the neuroendocrine system mediates the effect of loneliness on health [11].

For many years, researchers have indicated the universal presence of the experience of loneliness, which may affect 80% of the population aged 18–65 years [7,12,13]. However, it has become increasingly common among young people [14].

According to the World Health Organization, depression is one of the main causes of unfitness for work. There are 350 million people with depression worldwide. In Europe, there are 83 million people with depression, with Polish people accounting for approximately 10% (1.5 million), and 73% of them being women. However, it is estimated that the number of Polish people with depression may be twice as high [15]. A WHO study predicts that in 2030, depression will be one of the most commonly diagnosed diseases in the world [16]. The global economic effects of depression also need to be taken into account. The economic and social costs of treating mental disorders put a heavy burden on national health systems and economic development. The total cost of poor mental health in the 28 EU countries is estimated at over 4% of GDP. According to a report by the OECD and the European Commission, the costs of treating mental disorders in Poland account for 3.01% of GDP, with the total expenditure on health care estimated at 6.5% of GDP. In Poland, depressive states are the most frequently diagnosed disorders [17].

In May 2020, the United Nations issued a policy brief called “COVID-19 and the need for action on mental health”, with a view to mitigating mental health problems caused by the pandemic. A 2020 WHO report indicates that women are among the groups that are particularly susceptible to the negative impact of the COVID-19 pandemic in terms of mental health [18]. Current research supports the rationale for the monitoring of mental toughness in women, who are at particular risk of the negative effects of unpredictable events [19,20,21,22]. This study is important for the mental health assessment of women in Poland and for the identification of women who are particularly vulnerable to psychological consequences of the pandemic.

## 2. Aim of the Study

The aim of the study is assessing the prevalence of anxiety, depression, irritability, and a sense of loneliness among women during the COVID-19 pandemic.

## 3. Materials and Methods

### 3.1. Participants

The study was performed using Computer-Assisted Web Interviewing (CAWI) via a website. The study group was selected using random quota sampling. The study included 452 adult women interviewed on 6–12 October 2020 during the second wave of the SARS-CoV-2 pandemic in Poland. The structure of the study sample reflects the population structure of women in Poland regarding age, education, size of place of residence, and province; thus, the sample is representative in this respect. Before completing the questionnaire, the participants were asked for their consent to answer the questions regarding the issues covered by the survey. The study was conducted in line with the Declaration of Helsinki.

The subjects could withdraw their participation from the study at any point of completing the questionnaire. All answers were treated as strictly confidential, and the respondents were guaranteed full anonymity. The subjects completed the questionnaire using a dedicated website. The study was approved by the Bioethics Committee at the Medical University of Warsaw (approval No. AKBE/232/2020).

### 3.2. Measurement

#### 3.2.1. Socio-Demographic Questionnaire

The participants were asked to complete a socio-demographic questionnaire, which included questions regarding age, gender, marital status, education, employment status and place of residence. They were also asked to rate their financial situation. The subjects were asked whether they knew anyone with a positive test result for SARS-CoV-2 infection. The women self-rated their health as of the period before the pandemic. They were also asked to assess changes in their health during the pandemic. Furthermore, the subjects were also asked to indicate the presence of chronic diseases such as cardiovascular, respiratory, or endocrine diseases, cancer, kidney disease, or mental disorders.

#### 3.2.2. Hospital Anxiety and Depression Scale (HADS-M)

In order to evaluate anxiety and depression, a Polish version of the HADS-M, developed by Majkowicz et al., was used [23], which is a modified version of the Hospital Anxiety and Depression Scale (HADS) developed by Zigmond et al. The HADS contains 2 independent subscales: anxiety and depression, while two statements were added in the HADS-M to assess the level of irritability [24]. HADS-M is composed of 16 questions in total, with each of them scored 0 to 3. The maximum score separately for anxiety (7 questions) and depression (7 questions) is 21 points, and the maximum score for irritability (2 questions) is 6 points. A score of 0–7 indicates a lack of disorders, a score of 8–10 means a borderline state, and a score of 11–21 indicates the presence of disorders. The following interpretation was adopted in line with the questionnaire key for the anxiety and depression subscales: no disorders: 0–7 points, borderline states: 8–10 points, disorders: 11–21 points; the interpretation for the irritability subscale was: 0–2 points: no disorders, 3 points: borderline states, 4–6 points: disorders.

The Cronbach’s alpha for the internal consistency of the study tool with regard to the Hospital Anxiety and Depression Scale was α = 0.92.

#### 3.2.3. R-UCLA

In the study, the Polish version of the R-UCLA scale validated by Kwiatkowska et al. was used [25]. The original version of the scale is called UCLA LS and was developed by Russel et al. [26]. The scale is composed of 20 statements. The respondents select one out of 4 answers to describe the frequency which is true for them (1 = “I never feel this way”, 4 = “I often feel this way”). The maximum score is 80. The total score is a sum of scores from 3 subscales: belonging and affiliation, intimate others, and social others. The sense of loneliness was determined based on the Perry’s classification. Four levels of loneliness were defined: 65–80 points: high level of loneliness; 50–64 points: moderately high; 35–49 points: moderate and 20–34 points: low level of loneliness [27]. The Cronbach’s alpha for the internal consistency of our tool with regard to R-UCLA was α = 0.91.

#### 3.2.4. Statistical Method

The normality of the data distribution was determined using the Shapiro-Wilk test and homogeneity of variance was checked with the Levene’s test. Differences between groups were assessed with the Kruskal-Wallis test with a post hoc Dunn’s test for more than two groups being compared. For two comparison groups, the following were used: Student’s *t*-test for independent samples, the Cochran-Cox’s test for an unmet condition of homogeneity of variance, or Mann-Whitney U-test. Correlation analysis was performed using Pearson’s linear correlation coefficient r. The strength of correlations was assessed using J. Guilford’s classification [28]. Results for which the probability level met the condition *p* ≤ 0.05 were considered statistically significant. The calculations were performed using Statistica 10.0 StatSoft Poland.

## 4. Results

### 4.1. Characteristics of the Study Group

The study group included 452 women, 25% of whom were aged 60 years or more. The mean age of women in the study was 43 years. Rural inhabitants accounted for 36% and city dwellers for 64% of the study population. More than half of the women were professionally active (66.1%), while 23% were of an old age and incapacitated pensioners and 10% were students. In the study group, 29.7% of the women were in a relationship and 70.3% were single; 14% were in an unmarried relationship and 8% were separated from their spouses after a divorce. The smallest group were widows (5%). There were 63.6% of subjects who lived with their family and 25.8% who lived only with their spouse or partner. Respondents living alone accounted for 10% of the study group. The majority of women (41.5%) rated their financial situation as “neither good nor poor” and 6.1% as very poor. Among the women, 52.8% had secondary education.

### 4.2. Anxiety, Depression, and Irritability

On the HADS-M, the subjects scored M = 16.0, SD = 9.66. The mean scores were M = 7.90, SD = 4.76 for the anxiety subscale, M = 5.47, SD = 4.28 for the depression subscale, and M = 8.14, SD = 5.36 for the irritability subscale (Table 1).

**Figure 1:**
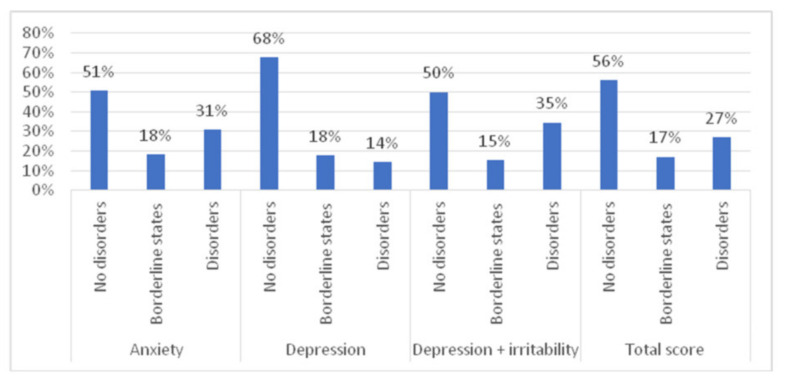
HADS-M scores in the study population of women.

On the anxiety subscale, disorders were found in 30.7% and borderline states in 18.3% of the subjects; on the depression subscale, disorders were demonstrated for 14.3% of the participants. The combined percentage of women with disorders on the depression and irritability subscales was 34.5%; borderline states were found in 15.4% of the subjects (Figure 1).

### 4.3. Loneliness

The highest values in the R-UCLA scale were obtained by the women in the total score subscale M = 40.29, SD = 10.82 (Table 2).

**Figure 2:**
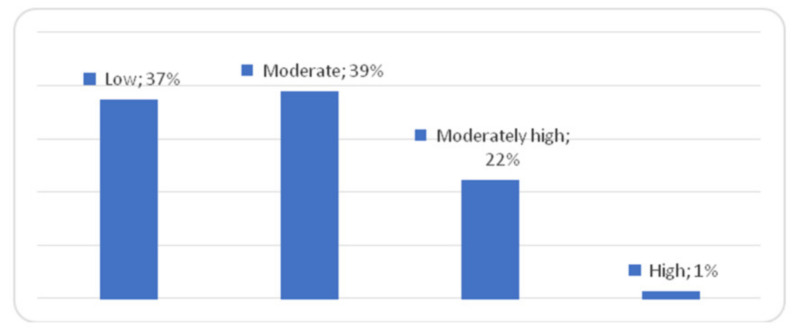
R-UCLA scores in the study population of women.

A low level of loneliness was found in 37.3% of the subjects, moderate in 38.9%, moderately high in 22.3% and high in 1.3% of the women (Figure 2).

### 4.4. Correlations between Loneliness and Anxiety, Depression, and Irritability

The highest strength of effect was found between the total R-UCLA score and total HADS-M score (r = 0.55) and individual R-UCLA components: belonging and affiliation (r = 0.54) and social others (r = 0.55) (Table 3).

A positive correlation was found between the two scales. Increasing loneliness scores were accompanied by increasing anxiety, depression, and irritability scores.

### 4.5. Sociodemographic Variables and the Severity of Anxiety, Depression, Irritation, and Loneliness

The youngest women scored the highest for anxiety and depression in HADS-M.

**Figure 3:**
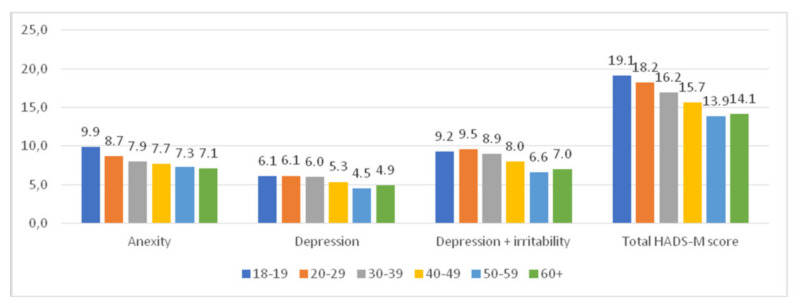
HADS-M score and the age of women.

Women aged ≤ 20–29 years were the dominant group in the subscale of depression and irritability. Women aged 60+ and 50–59 years scored the lowest in the anxiety subscale (Figure 3).

The highest total R-UCLA scores were obtained by women aged 18–19 and 20–29 years. Women aged 20–29 years scored the highest for loneliness in the social others subscale.

**Figure 4:**
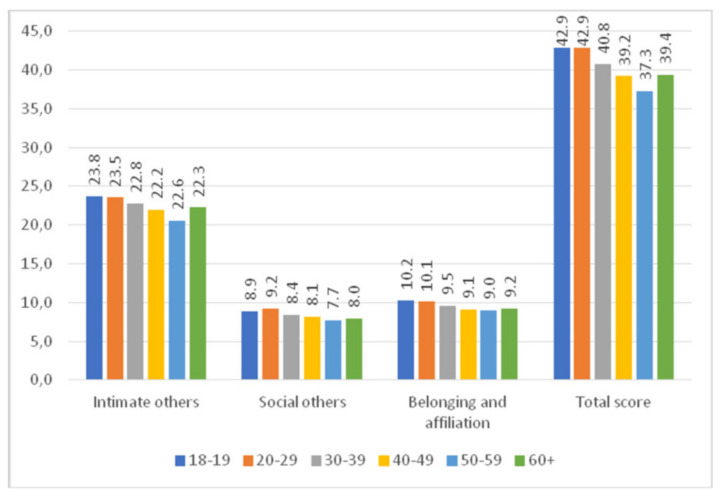
R-UCLA score and the age of women.

The highest and the lowest scores for loneliness in the intimate others subscale were obtained by women aged 18–19 years and 50–59 years, respectively (Figure 4).

We showed that anxiety, depression, and irritability decreased with age (*p* < 0.001; r = −0.17). Loneliness, as measured with R-UCLA, correlated with the marital status of the women in the study (*p* < 0.001). Women who were single were lonelier than those in relationships. HADS-M scores were significantly negatively correlated with the subjects’ self-rating of financial situation (*p* < 0.001). There was also a negative correlation between the financial situation of the women in the study and loneliness as measured with R-UCLA (*p* < 0.001). The women’s place of residence did not show any statistically significant correlation with R-UCLA or HADS-M scores in the study (Table 4).

**Figure 5:**
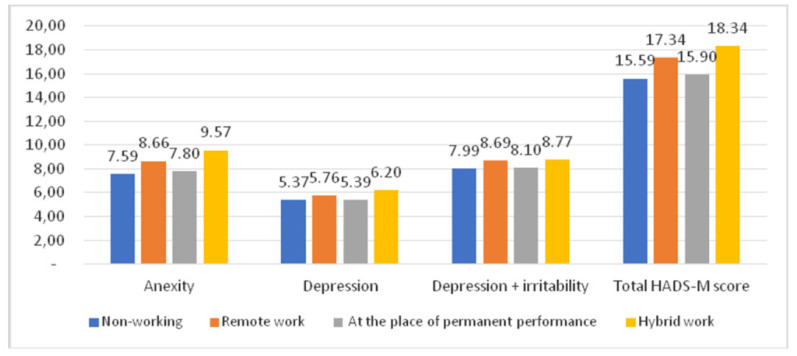
Place of work and the HADS-M score.

The group of surveyed women who had a hybrid work model, i.e., remote work combined with office time, scored the highest in all HADS-M subscales of anxiety, depression and irritability. Non-working women scored the lowest in all HADS-M subscales (Figure 5).

The highest loneliness score, as measured with R-UCLA, was found for intimate others in women with a hybrid work model. Women who worked from home scored the highest for loneliness in social others and belonging and affiliation.

**Figure 6:**
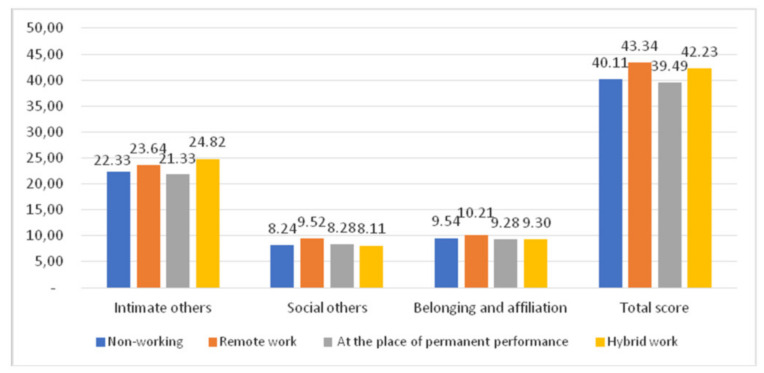
Place of work and the R-UCLA score.

The lowest scores for intimate others, belonging and affiliation, and total score in the R-UCLA scale were reported for women working in their workplace (Figure 6).

### 4.6. Health Variables and the Severity of Anxiety, Depression, Irritability, and Loneliness

Loneliness, as measured with R-UCLA, correlated with the marital status of the women in the study (*p* < 0.001). Women who were single were lonelier than those in relationships. HADS-M scores were significantly negatively correlated with the subjects’ self-rating of their financial situation (*p* < 0.001). There was also a negative correlation between the financial situation of the women in the study and loneliness, as measured with R-UCLA (*p* < 0.001). The women’s place of residence does not show any statistically significant correlation with R-UCLA and HADS-M scores in the study (Table 5).

Women who reported respiratory diseases displayed a significantly higher level of anxiety, depression, and irritability on the HADS-M (*p* = 0.024). A higher level of loneliness in the R-UCLA was found in subjects with endocrine diseases (*p* = 0.041). The presence of mental disorders among the women in the study was positively correlated with anxiety, depression, and irritability (HADS-M), as well as with loneliness (R-UCLA) (*p* < 0.001) (Table 6).

Women who stated that they did not know anyone with a positive test result for SARS-CoV-2 displayed a higher sense of loneliness on R-UCLA than women who did know someone with a positive test result. Anxiety and depression as measured by HADS-M did not significantly correlate with knowing someone with a positive test result for SARS-CoV-2 (Table 7).

## 5. Discussion

In March 2020, a statement was published urging for research to be conducted on the impact of the COVID-19 pandemic on mental health among particularly vulnerable populations and groups [29]. Wenham et al. and Holmes et al. point to the fact that public health action has usually given marginal attention to the problem of gender-related consequences of epidemics. The authors believe that the same is the case for COVID-19. The authors emphasize that the degree to which disease outbreaks have a differential impact on women and men is the basis for understanding the primary and secondary health risks for various individuals and communities, and for developing effective and fair policies and interventions [29,30].

Many studies published to date point to negative social and economic consequences of the current stay-at-home orders and the COVID-19 pandemic itself. These contribute to adverse psychological effects, including deterioration of loneliness, depression, anxiety, and financial concerns [31,32]. The COVID-19 pandemic is a substantial threat both for physical and mental health since it can lead to psychological stress associated with economic crisis, threat of unemployment, or fear of losing family members [33].

Mental health during the COVID-19 pandemic is an important research area; for this reason, our study aimed to measure psychological well-being (level of depression, anxiety, and loneliness) in a sample of Polish women. We wished to focus on problems that can occur in women during a period of social isolation, since they are as important as the fight against the disease itself, and the consequences may be serious in the long term. The aim of the present study was to estimate the prevalence of anxiety, depression, irritability, and sense of loneliness among women during the COVID-19 pandemic. The study was conducted during the second wave of the pandemic in Poland, which was much more dynamic than the first one. Moreover, the number of deaths and ventilator occupancy rate were increasing at the time. The number of Polish people infected with SARS-CoV-2 is 2,642,242.

Numerous studies have demonstrated that symptoms of depression and anxiety are highly prevalent during the COVID-19 pandemic [34,35,36]. Studies conducted in different parts of the world also identified a higher severity of mental health problems among women than men during the pandemic [37,38,39]. However, in a study conducted in China, Wu et al. observed a higher level of depression and anxiety among men [40]. During the initial period of the COVID-19 pandemic, Roob et al. conducted a study among 7127 London inhabitants aged over 50 years, with women accounting for 54.1% of the study population. They observed deterioration of depressive disorders in 17.3% and anxiety in 16.5% of women, which are lower results than the ones in the present study [5].

In longitudinal studies conducted by Freeman et al. and Lorant et al. in the period before the pandemic, an association between symptoms of depression and anxiety and the subjects’ socioeconomic situation was demonstrated [41,42]. Our research shows that feelings of loneliness among women in Poland during the pandemic was also associated with socioeconomic status. Loneliness was more common among women who assessed their financial situation as bad and those who were lonely. Chronic loneliness, as research shows, leads to many mental disorders, such as self-destructive behavior [43]. Therefore, we believe that prophylaxis aimed at preventing the negative effects of loneliness should be directed at women.

This is corroborated by the findings from our study. The socioeconomic situation had an impact on the sense of loneliness of women taking part in our study. In addition, loneliness was more common among women who rated their financial situation as poor and were single. Death of a spouse and divorce were observed to be a risk factor for mental health deterioration in similar general population studies during the COVID-19 pandemic in Spain (n = 3055) [44] and China (n = 1060) [45]; however, the impact of gender was not investigated. One may expect that an increased level of loneliness is inherent in living alone and without a partner, particularly in times of social and physical distancing.

Losda-Baltar et al. indicate that loneliness among young adults, including women, during the COVID-19 pandemic is higher than among older women [46]. Our study also confirmed the greatest sense of loneliness among the youngest groups of women. The WHO report shows that loneliness and social isolation are a frequent predictor of suicide attempts in the youngest age groups of women [47]. This confirms our belief that systematic therapeutic activities are needed in this group of women. Social media can become an effective tool to fight loneliness.

The results of other researchers, Lorena García-Fernández et al., indicate that older women coped better with the challenges of accustoming oneself to the new situation associated with the COVID-19 pandemic [48]. It might be assumed that during an epidemic, older women have more resources to cope with the changes associated with the situation (more life experience) and may have more emotional distance to it. In addition, it seems that the fulfilment of developmental needs of such women is limited to a lower extent with the epidemic than that of young women [49].

The SHARE research project, which was conducted in 12 European countries, assessed the level of loneliness in the population aged over 65 years. The association between loneliness and various socioeconomic factors and subjective health status was significantly higher in women. Loneliness was the highest for Spain, France, and Greece (OR (95% CI): 2.00 (1.23–3.25), 2.39 (1.24–4.60) and 1.71 (1.12–2.62), respectively). This longitudinal study conducted for two decades showed that the sense of loneliness is more common among older women in southern Europe than northern Europe [50]. According to the results of our study with regard to the sense of loneliness during the COVID-19 pandemic, high and moderately high levels of loneliness were present in 2 out of 3 women in total.

In a study conducted among elderly Germans, 13.1% of the subjects declared feeling lonely, with the figure for women being 16.3%. Lower loneliness scores were obtained for older participants compared to younger ones. However, it needs to be noted that loneliness was nearly twice as high in women than in men. The study was conducted during the initial period of the pandemic in Germany [51].

There was a significant negative association between subjective loneliness and deterioration in both depression (OR: 17.24, 95% CI: 13.20, 22.50) and anxiety (OR: 10.85, 95% CI: 8.39, 14.03) [37]. This is corroborated by research in other countries [34,52]. Such results were also obtained in various age groups in the United Kingdom coming from a general cohort of 17,452 individuals with women accounting for 41.8% of the study population (13.6%, 95% CI: 13.4–13.8) [53]. Similar findings were seen by researchers in Germany [54], Spain [44,55,56,57], Italy [35], and Iran, where anxiety levels were also higher in women (95% CI: 0.1, 81.36, *p* < 0.001), who accounted for 65.8% of the whole study population [34,58]. Our study fully corroborates this phenomenon. Referring to the 2017 report Depression and Other Common Mental Disorders Global Health Estimates, World Health Organization in Poland, depressive and anxiety disorders were diagnosed in 5.1% and 3.9% of Polish people, respectively. The other shows that women over 50 years of age in Poland are at the highest risk of depressive disorders [59].

This relationship is also confirmed by our study. It was women in the youngest age groups who showed the highest levels of depression and anxiety. We believe this may have economic consequences. In Poland, young people are usually employed under civil law contracts, which could make it easier to lose employment during the pandemic. Due to the closure of nurseries, kindergartens, and schools, women were on childcare leave. A significant proportion of young women work in the tourism or catering industries, which have been most affected by the pandemic.

In the majority of the aforementioned studies, it was observed that younger women had a higher level of depression and anxiety than women over 50 years of age. This relationship is also confirmed by our study. In some of these studies, the following research tools were used to measure depression and anxiety, respectively: Patient Health Questionnaire-9 (PHQ-9) and Generalized Anxiety Disorder 7-item (GAD-7). In a study by Jia et al. in a large cohort of adults from the United Kingdom (N = 3097, aged ≥ 18 years, including 2618 women) concerning the impact of the COVID-19 pandemic on mental health, PHQ-9 was used as the research tool. In this study, the mean results for depression (=7.69; SD = 6.0), stress (=6.48; SD = 3.3), and anxiety (=6.48, SD = 3.3) were significantly higher than normal population values (*p* < 0.001 for all). It is worth taking note of results which show that women and young individuals are at a particular risk of depression alone [9].

Similarly, Losda-Baltar et al. indicate that loneliness among young adults, including women, during the COVID-19 pandemic is higher than among older women [46]. A preliminary report from a longitudinal study conducted during the COVID-19 pandemic in Poland by Gambin et al. in a cohort of 1179 individuals (with women accounting for 49.7%) shows that individuals over 64 years of age have a significantly lower level of depression than subjects aged 18–24 and 25–35 years, and a lower level of anxiety than those aged 18–24 years. In addition, the age of the subjects was negatively correlated with the symptoms of severity for both depression (r = −0.231; *p* < 0.001) and anxiety (r = −0.180; *p* < 0.001) [60]. This relationship is also confirmed by our study.

In our study, older women showed lower intensity of depression and anxiety symptoms. This may be due to greater economic stability or life experience. However, we can see the need for further research and close monitoring of the psychosocial functioning of senior women, especially in the event of a prolonged pandemic.

In a study by Lee et al. conducted among young adults aged 22–29 years living in South Korea, with women accounting for 60.7% of the study population, an increase in the level of loneliness was observed. Changes in loneliness also had a clear effect on the increase in depression, but not in anxiety [61]. In our study, the sense of loneliness correlated positively with depression, anxiety, and irritability (*p* < 0.001 for all). It is worth taking note of the fact that during the first wave of the pandemic, South Korea was able to control the spread of COVID-19 effectively without nationwide lockdowns or drastic efforts to ensure social distancing.

Older women rate their subjective well-being as poorer, with an increasing sense of loneliness and a number of chronic diseases [62]. The most important risk factors for death due to COVID-19 include advanced age and comorbidities [63,64,65]. Their presence may exacerbate depression and anxiety [66]. We obtained similar findings in our study. There is a significant correlation between the level of loneliness and the prevalence of depressive symptoms and the presence of certain chronic diseases (endocrine diseases, kidney disease, and mental disorders). Mental disorders are the strongest predictor for loneliness, anxiety, depression, and irritability in the study population of women. In a study conducted in Turkey during the COVID-19 pandemic, 23.6% of the population scored higher than the cut-off point for depression, and 45.1% scored higher than the cut-off point for anxiety. In this study, female gender, psychiatric disorders, psychological disturbances, and chronic diseases were considered to be risk factors for anxiety [67]. A study by Feter et al. covered a cohort of adult Brazilians (n = 2314) aged from 31 to 59 years, with women accounting for 76.6% of the study population. In this study, female gender (PR: 1.27; 95% CI: 1.11 to 1.45), chronic diseases (PR: 1.17; 95% CI: 1.05 to 1.29), and poor financial situation (PR: 1.15; 95% CI: 1.04 to 1.28) were predictors of anxiety and depression. A study by Feter et al. shows that young women up to 30 years of age have the highest levels of anxiety and depression [68]. In our study, chronic diseases and poor financial situations correlated positively with anxiety and depression. In China, where the SARS-CoV-2 pandemic began, Liu et al. identified female gender as the strongest predictor of stress after the pandemic [39].

To the best of our knowledge, our study has been the first in Poland and perhaps in the whole world to ask women about their self-rating of physical and mental health in the period before the pandemic and ask them to assess the change in their health during the pandemic. The women’s self-rating of mental health revealed a strong association with loneliness in all R-UCLA subscales and with depression, anxiety, and irritability in HADS-M, unlike physical health self-rating. During the pandemic, the highest change in the self-rating of physical and mental health in the study population of women was seen for anxiety and depression. However, we cannot establish any cause-and-effect relationships due to the cross-sectional nature of the study.

## 6. Conclusions

We found anxiety and depression disorders in the study population of women. The affected individuals mainly included women who lived alone, had poorer self-rating of financial situation, lower subjective health rating and certain chronic diseases. Two in three women experienced loneliness. These were mainly women who were single, lived alone, rated their financial situation and physical and mental health as poor, and had certain chronic conditions, particularly mental disorders.

Women aged 18–29 years showed the highest levels of anxiety, depression, irritability, and loneliness.

Further research, including longitudinal studies on the mental health of women during the COVID-19 pandemic, is needed.

In our opinion, women of all ages should be assessed in terms of mental functioning, and intervention planning should be age appropriate. Therapeutic classes for women attending secondary schools or higher education institutions may take place at school or university and should be implemented systemically. We consider it necessary to plan preventive interventions to help young adult women cope with the various difficulties encountered during the pandemic. We also see the need to implement therapeutic measures during and after the pandemic in order to alleviate its psychological consequences for women.

## Figures and Tables

**Table 1:** HADS-M scores for anxiety, depression, and irritability and scores for the sense of loneliness: descriptive statistics.

	Variable	M	Median	Min.	Max.	SD
HADS-M	Anxiety	7.90	7.00	0.0	21.0	4.76
	Depression	5.47	5.00	0.0	20.0	4.28
	Depression + Irritability	8.14	7.50	0.0	26.0	5.36
	Total score	16.0	15.00	0.0	47.0	9.66

**Table 2:** R-UCLA scores for loneliness: descriptive statistics.

	Variable	M	Median	Min.	Max.	SD
R-UCLA	Belonging and affiliation	9.46	9.00	5.00	19.00	2.86
	Intimate others	22.48	22.00	10.00	40.00	6.93
	Social others	8.34	8.00	5.00	20.00	3.05
	Total score	40.29	39.00	20.00	69.00	10.82

**Table 3:** Correlations between loneliness and anxiety, depression, and irritability.

HADS-M	R-UCLA			
	Intimate Others	Social Others	Belonging and Affiliation	Total Score
Anxiety	r = 0.46	r = 0.38	r = 0.35	r = 0.49
	*p* < 0.001	*p* < 0.001	*p* < 0.001	*p* < 0.001
Depression	r = 0.48	r = 0.47	r = 0.42	r = 0.55
	*p* < 0.001	*p* < 0.001	*p* < 0.001	*p* < 0.001
Depression + irritability	r = 0.49	r = 0.45	r = 0.39	r = 0.54
	*p* < 0.001	*p* < 0.001	*p* < 0.001	*p* < 0.001
Total HADS-M score	r = 0.50	r = 0.44	r = 0.39	r = 0.55
	*p* < 0.001	*p* < 0.001	*p* < 0.001	*p* < 0.001

**Table 4:** Sociodemographic variables with regard to HADS-M and R-UCLA scores.

Parameter	% of All Subjects	HADS-M			R-UCLA		
		M	SD	t/H/r	M	SD	t/H/r
Age	100.00	16.5	9.66	*p* < 0.001 r = −0.17	40.29	10.82	*p* = 0.004 r = −0.13
**Marital status**							
In a relationship	70.3	10.64	9.50	*p* = 0.259 t = 1.11	39.05	10.24	*p* < 0.001 t = 3.28
Single	29.7	16.76	9.79		42.90	11.62	
**Education**							
Primary/vocational/lower secondary	30.2	18.92	11.50	H = 6.95 *p* = 0.073	41.38	10.77	H = 1.24 *p* = 0.742
Secondary/post-secondary	52.8	15.43	9.74		40.64	11.11	
Higher	17.0	15.06	15.06		38.96	9.94	
**Employment status**							
Old-age pensioner/incapacitated pensioner	25.3	14.12	10.32	H = 7.36 *p* = 0.061	39.70	10.50	H = 3.56 *p* = 0.312
Unemployed	16.3	16.41	9.00		39.00	9.89	
Employed	48.0	16.41	9.63		40.44	11.40	
Students	10.4	17.18	9.60		43.25	10.85	
**Place of residence**							
Rural area	38.0	16.42	9.67	r = 0.050 *p* = 0.279	39.41	9.99	r = 0.035 *p* = 0.445
City of up to 20,000 inhabitants	10.0	18.22	10.90		41.12	11.69	
City of 21,000–50,000 inhabitants	13.5	14.25	9.28		39.01	9.94	
City of 51,000–100,000 inhabitants	9.2	15.26	9.82		39.93	11.07	
City of 101,000–200,000 inhabitants	9.0	15.76	9.33		39.66	10.89	
City of 201,000–500,000 inhabitants	8.3	14.93	10.68		41.12	13.26	
City of over 500,000 inhabitants	12.0	16.06	8.79		42.51	11.85	
**Person with whom one currently resides**							
Alone	10.6	13.74	10.66	H = 7.95 *p* = 0.018	42.06	12.12	H = 1.40 *p* = 0.494
With spouse/partner only	25.8	15.01	9.89		39.56	10.27	
With family (children, relatives)	63.6	16.84	9.30		40.05	10.67	
**Financial situation rating**							
Very good	3.7	14.47	10.45	r = −0.280 *p* < 0.001	34.94	11.87	r = −0.194 *p* < 0.001
Quite good	34.4	13.57	8.74		38.80	10.42	
Neither good nor poor	41.5	15.49	8.75		40.20	10.55	
Quite poor	14.3	20.07	10.53		42.43	10.61	
Very poor	6.1	27.58	8.53		48.95	11.00	

r—Pearson’s correlation coefficient, t—Student’s *t*-test, H—Kruskal-Wallis test; *p*—statistical significance.

**Table 5:** Self-rating of health status before the pandemic and assessment of health changes during the pandemic regarding R-UCLA and HADS-M.

Pair of Variables	Spearman Rank Correlation Test			
	Self-Rating of Health Status before the Pandemic		Self-Rating of Change in Health during the Pandemic	
	R Spearman	*p*	R Spearman	*p*
Physical health and anxiety	0.29	0.000	0.12	0.012
Physical health and depression	0.32	0.000	0.11	0.015
Physical health and depression + irritability	0.30	0.000	0.12	0.013
Physical health and total HADS-M score	0.31	0.000	0.12	0.011
Physical health and intimate others	0.20	0.000	0.12	0.008
Physical health and social others	0.20	0.000	0.05	0.295
Physical health and belonging and affiliation	0.20	0.000	0.09	0.065
Physical health and total score	0.23	0.000	0.10	0.031
Mental health and anxiety	0.55	0.000	0.36	0.000
Mental health and depression	0.56	0.000	0.32	0.000
Mental health and depression + irritability	0.56	0.000	0.30	0.000
Mental health and total HADS-M score	0.58	0.000	0.34	0.000
Mental health and intimate others	0.37	0.000	0.15	0.000
Mental health and social others	0.32	0.000	0.15	0.000
Mental health and belonging and affiliation	0.32	0.000	0.20	0.000
Mental health and total score	0.39	0.000	0.18	0.001

R—Spearman rank test, *p*—statistical significance.

**Table 6:** Presence of chronic diseases regarding HADS-M and R-UCLA scores.

Parameter	% Yes	HADS-M				R-UCLA			
		M	SD	Z	*p*	M	SD	Z	*p*
Cardiovascular diseases	2.7	16.32	11.03	0.151	0.879	40.25	11.53	0.220	0.825
Respiratory diseases	10.5	19.67	11.17	2.153	0.024	41.81	11.25	0.890	0.373
Endocrine diseases	24.0	17.08	10.43	0.745	0.456	42.51	11.20	2.043	0.041
Cancer	3.4	17.53	11.17	0.551	0.581	40.33	10.32	0.082	0.934
Kidney disease	3.9	19.23	9.58	1.486	0.137	43.58	10.45	1.295	0.194
Mental disorders	5.8	27.00	9.75	5.099	0.000	48.76	10.82	3.685	0.001
None of the above	49.7	17.31	10.25	2.263	0.023	41.27	10.91	1.837	0.066

Z—Mann-Whitney U-test, *p*—statistical significance.

**Table 7:** Knowing someone with a positive test result for SARS-CoV-2 regarding HADS-M and R-UCLA scores.

Variables	M Yes	M No	SD	t	*p*
Anxiety	7.99	7.84	4.87	0.28	0.774
Depression	5.27	5.50	4.08	0.49	0.624
Depression + irritability	8.12	8.11	5.37	0.01	0.991
Total HADS m score	16.10	15.95	9.77	0.14	0.883
Intimate others	21.64	22.54	6.34	1.21	0.224
Social others	7.88	8.54	2.82	2.02	0.043
Belonging and affiliation	8.82	9.72	2.91	2.88	0.004
Total R-UCLA score	38.34	40.81	10.61	2.08	0.037

t—Student’s *t*-test; *p*—statistical significance.

## Data Availability

The datasets generated and/or analyzed during the current study are not publicly available due to confidentiality, but data is accessible from the authors on reasonable request.
